# Synthesis of TiO_2_-incorporated activated carbon as an effective Ion electrosorption material

**DOI:** 10.1371/journal.pone.0282869

**Published:** 2023-03-23

**Authors:** Nasser A. M. Barakat, Yasmin T. Sayed, Osama M. Irfan, Marawa M. Abdelaty

**Affiliations:** 1 Faculty of Engineering, Chemical Engineering Department, Minia University, El-Minia, Egypt; 2 Department of Mechanical Engineering, College of Engineering, Qassim University, Buraydah, Saudi Arabia; University of Sharjah, UNITED ARAB EMIRATES

## Abstract

Efficient, chemically stable and cheap materials are highly required as electrodes in the ions-electrosorption-based technologies such as supercapacitors and capacitive deionization desalination. Herein, facile preparation of titanium oxide-incorporated activated carbon using cheap precursors is introduced for this regard. The proposed material was synthesized using the solubility power of the subcritical water to partially dissolve titanium oxide particles to be adsorbable on the surface of the activated carbon. Typically, an aqueous suspension of commercial TiO_2_ particles (P25) and activated carbon was autoclaved at 180°C for 10 h. The physiochemical characterizations indicated high and uniform distribution of the inorganic material on the surface of the activated carbon. The ionic electrosorption capacity was highly improved as the specific capacitance increased from 76 to 515 F/g for the pristine and modified activated carbon, respectively at 5 mV/s in 0.5 M sodium chloride solution. However, the weight content of titanium oxide has to be adjusted; 0.01% is the optimum value. Overall, the study introduces novel and simple one-pot procedure to synthesis powerful titanium oxide-based functional materials from cheap solid titanium precursor without utilization of additional chemicals.

## Introduction

Electrosorption capacity is the main feature for the electrode materials used in the most important advanced energy storage devices; supercapacitors. Moreover, it is highly recommended electrode material characteristic in the capacitive deionization desalination units. Supercapacitors are high-capacity energy storage devices can store and deliver energy at higher rates than batteries due to a simple charge separation mechanism at the electrode-electrolyte interface [1]. The underlying principle behind energy storage is the development of an electric double layer (EDL) as ions are adsorbed on the electrode surface. Similarly, the capacitive deionization (CDI) water purification technology is based on the electric double-layer capacitor [2]. Typically, in the CDI, salt ions in the input water are electrosorbed by the oppositely charged electrodes when an external electrical potential is applied, and when the voltage is reduced, the ions are ejected and the electrodes are regenerated enabling a continuous desalination process [3]. Because of the electrosorption feature, carbon exhibits a capacitive response owing to the buildup of charges in the EDL. Many research are aiming to replace carbon electrodes with pseudocapacitive (oxides or nitrides) or redox active materials that have a greater capacitance due to their charge storage mechanism [4]. Although these materials distinctly enhance the specific capacitance, the high electrical resistance results in a reduction in power density and a reduction in cycle life [5].

As a result, using carbonaceous materials as a matrix for the electrode and compositing it with pseudocapacitors (PCs) is an interesting strategy. Among the used carbonaceous supports, activated carbon (AC) drew the maximum attention due to many advantages compared the nano-scale carbons (e.g. carbon nanotubes, graphene, carbon dots, carbon nanofibers … etc.) including very low cost, availability in big amounts and simple preparation technologies with high yield. Indeed, compared to the nano-scale carbons, the surface area and electron transfer properties of the activated carbon are low, but, from the technological application’s point of view, the differences are not high and do not recompense the aforementioned advantages. Moreover, in the field of the electrochemical devices, activated carbon shows very acceptable specific surface area, good electrochemical stability, good conductivity and high supercapacitor cycle life [6].

As pseudocapacitors electrode materials, transition metal oxides such as titanium oxide (TiO_2_) [7,8], nickel oxide (NiO) [9], Manganese oxide (MnO_2_) [10], cupper monoxide (CuO) [11], cobalt oxide (Co_3_O_4_) [12] and vanadium oxide (V_2_O_5_) [13] have been widely investigated. Due to its low cost, high durability, eco-friendly nature, and natural abundance. TiO_2_ is one of the most promising candidate materials among them. Therefore, TiO_2_/AC composites are the most reported ones [14,15]. The electrical double-layer capacitance and electrosorption capacity of the AC electrode were found to be improved when TiO_2_ was added [16]. It was theorized that the influence of the single-direction polarity of TiO_2_ particles [17] or the decrease of physical adsorption of the AC surface through the interaction of TiO_2_ precursor with the polar group of AC caused the augmentation [18].

There are several methodologies have been proposed to prepare TiO_2_/AC composites including sol-gel [19], microwave [6,20], chemical co-precipitation [21], dip-hydrothermal [22], coating [23], pyrolysis [24], molecular adsorption-deposition [25], evacuation [26] and normal hydrothermal [27]. However, to the best of our knowledge, expensive TiO_2_ precursors were utilized in these synthesis processes e.g. titanium isopropoxide, titanium isobutoxide, titanium chloride … etc. Compared to these expensive liquid precursors, titanium oxide nanoparticles (P25) is highly available with very lower cost. Consequently, preparation of a worthwhile TiO_2_/AC composite from these cheap P25 and activated carbon precursors will be highly appreciable strategy.

Water that retains its liquid condition at temperatures between its boiling point of 100°C and its critical point of 374°C under proper pressure is referred to as subcritical water. It’s also known as pressurized hot water or superheated water. Subcritical water has two significant properties at these conditions: first, its dielectric constant falls, allowing it to function as a solvent for hydrophobic substances, and second, large magnitude of ions are produced at increased temperatures. As a result, water has wonderful properties in subcritical circumstances and may be used in a variety of applications [28,29].

Herein, TiO_2_-incorporated AC is introduced as a valuable EDLs/PCs composite with distinctly high supercapacitance. It was aimed that to prepare this efficient functional material to be utilized as an electrode in the supercapacitors from cheap precursors and simple synthesis strategy. As aforementioned, most of the reported TiO_2_‒based composites were based on liquid titanium precursors. However, to prepare TiO_2_‒based functional material, the liquid precursors are very expensive compared to rutile P25. For instance, the price of titanium isopropoxide is 60 USD/100 ml, titanium chloride is 51 USD/100 ml and titanium butoxide is 82 USD/250 ml. However, rutile P25 price is ~ 2.5 USD/kg. Accordingly, rutile P25 was selected as a precursor in this study. The proposed functional material was prepared by exploiting the ability of the subcritical water in partial dissolution of P25 to be adsorbable on the surface of the AC. Consequently, based on the utilized physicochemical analyses, AC could be decorated by a thin layer of TiO_2_. The electrochemical measurements showed a high increase in the specific capacitance of the prepared composite compared to the pristine AC.

## Materials and method

### 2.1 Preparation

Titanium oxide NPs (P25; Sigma-Aldrich) and commercial activated carbon (AC; CEP-21K, PCT Co., Korea) were utilized without any treatment. A 100 ml aqueous slurry, containing 0.1 g AC and a certain amount of P25, was first sonicated for 1 h to ensure high homogeneity, then was autoclaved in a hydrothermal reactor at 180°C for 10 h. Later on, the resulting solution was filtered and the remaining solid material was washed with H_2_O many times till the filtrate became clear. To optimize the inorganic oxide content, several samples were prepared; 0.007, 0.01, 0.02, 0.05 and 0.1 wt.% TiO_2_ with respect to the dry AC. In other words, the initial mixture contains 0.007, 0.01, 0.02, 0.05 and 0.1 g P25 per 1 g AC. The specific capacitance was estimated from the following [30]:

$$C=\frac{1}{2vm}\int \frac{I}{V}dV$$ (1)

Where ***C*** is the specific capacitance (F/g), *m* is the mass of the active material (g), *v* is the scan rate (V/s), *V* and *I* are the applied voltage (V) and the obtained current (A), respectively. Applying the numerical integration, the above equation can be rewritten as follow [31]:

$$C=\frac{1}{2vm}\sum_{n=1}^{n=N-1}\frac{\left(V_{n+1}-V_{n})\times (I_{n+1}+I_{n}\right)}{\left(V_{n+1}+V_{n}\right)}$$ (2)

*N* is the number of points in the CV cycle.

### 2.2 Characterization

Rigaku X-ray diffractometer (XRD, Rigaku, Japan) was utilized to investigate the chemical composition of the prepared material, while the morphology and the elemental analysis were explained by SEM (scanning-electron-microscope; JEOL-JSM-5900, Japan). Cyclic voltammetry analyses were carried out in three electrodes cells operated by the VersaStat4 equipment. Platinum wire, glassy carbon, and silver/silver chloride (Ag/AgCl) electrodes were utilized as counter, working, and reference electrodes, respectively, in a traditional three electrodes cell. The working electrode was made by dissolving 0.002 g of the prepared material in a solution of 5 μL Nafion solution (5 wt%) and 400 μL isopropanol. 15 μL of the produced slurry were poured over the working electrode’s active region. The electrode was then dried at 80°C. It is worth mentioning that the introduced physicochemical characterizations belong to the 0.1% sample because the results were clear and informative. On the other hand, the electrochemical measurements were conducted for all samples.

## 3. Results and discussion

**Fig 1:**
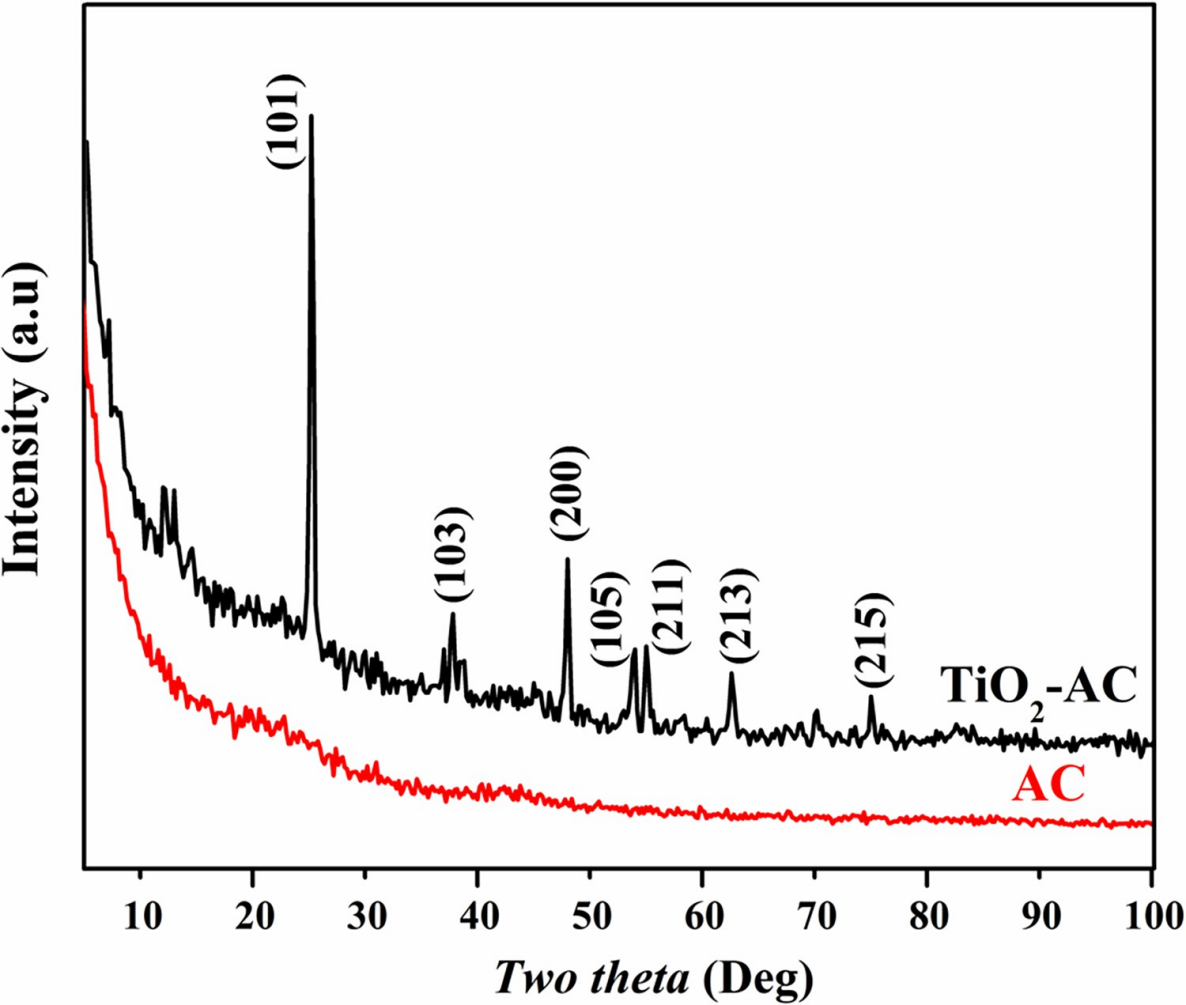
XRD patterns for the pristine and TiO_2_-containing 0.1% activated carbon.

X-ray diffraction is a trustable technique detecting the crystalline materials. Fig 1 displays the obtained patterns for the pristine and treated AC. As shown, due to the amorphous structure of the utilized commercial AC, no diffraction peaks could be obtained in the corresponding pattern. However, for the treated material, anatase TiO_2_ could be detected due to the appearance of strong diffraction peaks at 2*θ* values of 25.15°, 36.54°, 47.95°, 53.89°, 55.07°, 62.40° and 75.00° corresponding to the crystal planes (101), (103), (200), (105), (211), (213) and (215), respectively [JCPDS card # 21–1272]. However, no peaks corresponding to other TiO_2_ phases (e.g. rutile) could be detected. Beside the mentioned peaks there are other peaks can be observed at 2*theta* range of 11° to 17° which can be assigned to hydrogen titanate (H_2_Ti_3_O_7_) compound according to JCPDS database # 41–0192. However, according to the peaks intensities, a trace amount of H_2_Ti_3_O_7_ is detected. Formation of the H_2_Ti_3_O_7_ draws the attention toward the proposed formation mechanism of TiO_2_ nanotubes using the alkali hydrothermal treatment of TiO_2_ nanoparticles [32]. Simply, a disordered phase from H_2_Ti_3_O_7_ nanocrystals is formed due to peeling off the solid NPs by the used strong alkali solution, and then the nanocrystals are assembled to form the nanotubes [33]. Consequently, it can be concluded that the subcritical water does have the power of the partial dissolution of the titanium oxide nanoparticles to produce the H_2_Ti_3_O_7_ nanocrystals.

**Fig 2:**
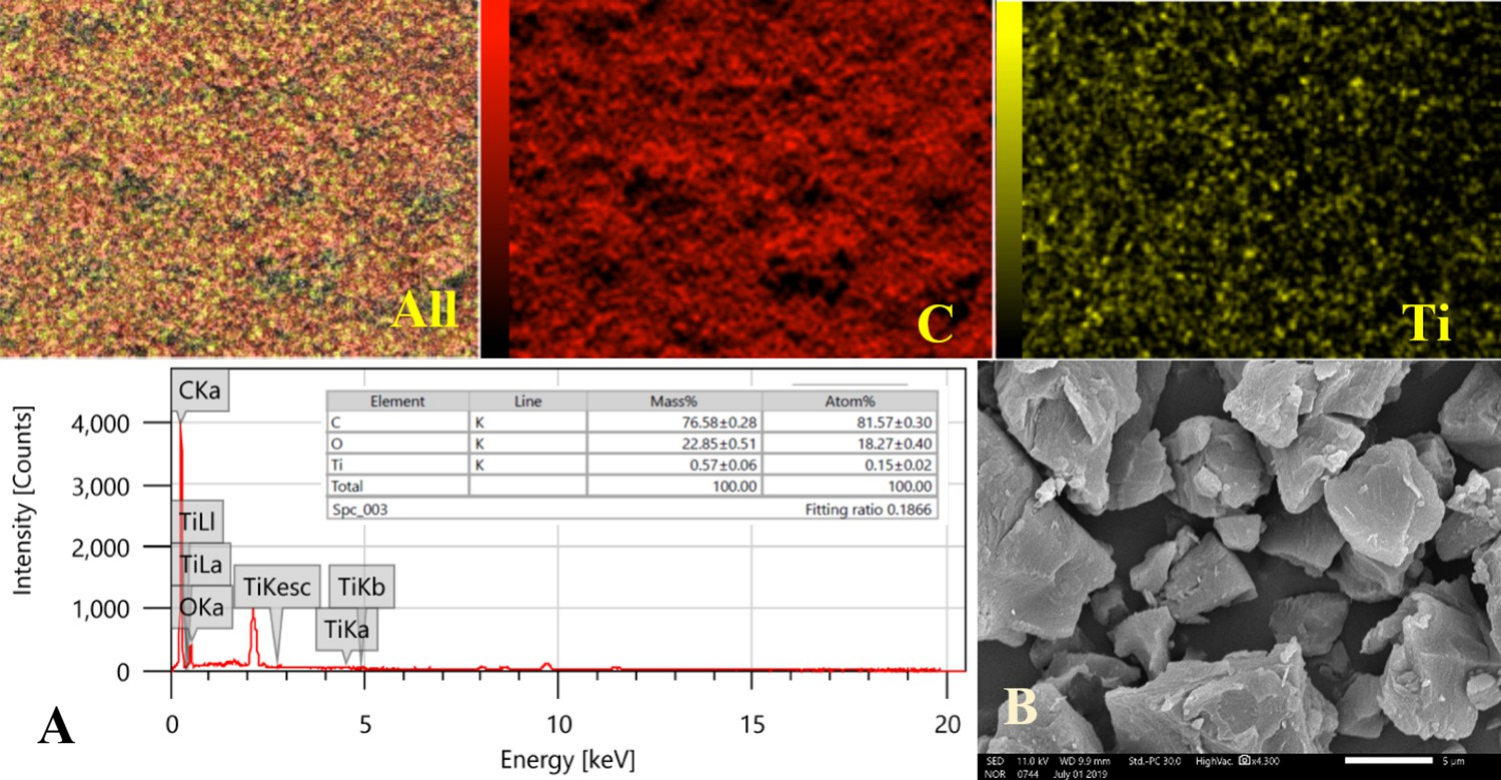
The upper images show elemental mapping analyses for the prepared TiO_2_- incorporated AC with individual distribution of titanium and carbon. The lower panels display EDX results; (A), and SEM image for the composite sample; (B), the scale bar represents 5 μm.

The upper images in Fig 2 display the elemental mapping analyses, as can be concluded, from the titanium metal and carbon distributions, the metal has a uniform distribution on the surface of the activated carbon. Moreover, the two elements have a similar distribution. Therefore, it can be concluded that the formed H_2_Ti_3_O_7_ nanocrystals were adsorbed on the surface of the AC, later on most of these nanocrystals were decomposed to anatase phase which was scientifically proved [33,34]. Fig 2A shows the EDX analysis which further confirmed the formation of TiO_2_-incoporated AC. Fig 2B represents SEM image of the produced TiO_2_-incoporated AC (0.1% sample). As shown, only the AC particles can be shown while the used TiO_2_ is absent. From the elemental mapping of titanium (Ti panel in Fig 2), it can be concluded that TiO_2_ is existing in the form of a thin on the surface of the AC.

**Fig 3:**
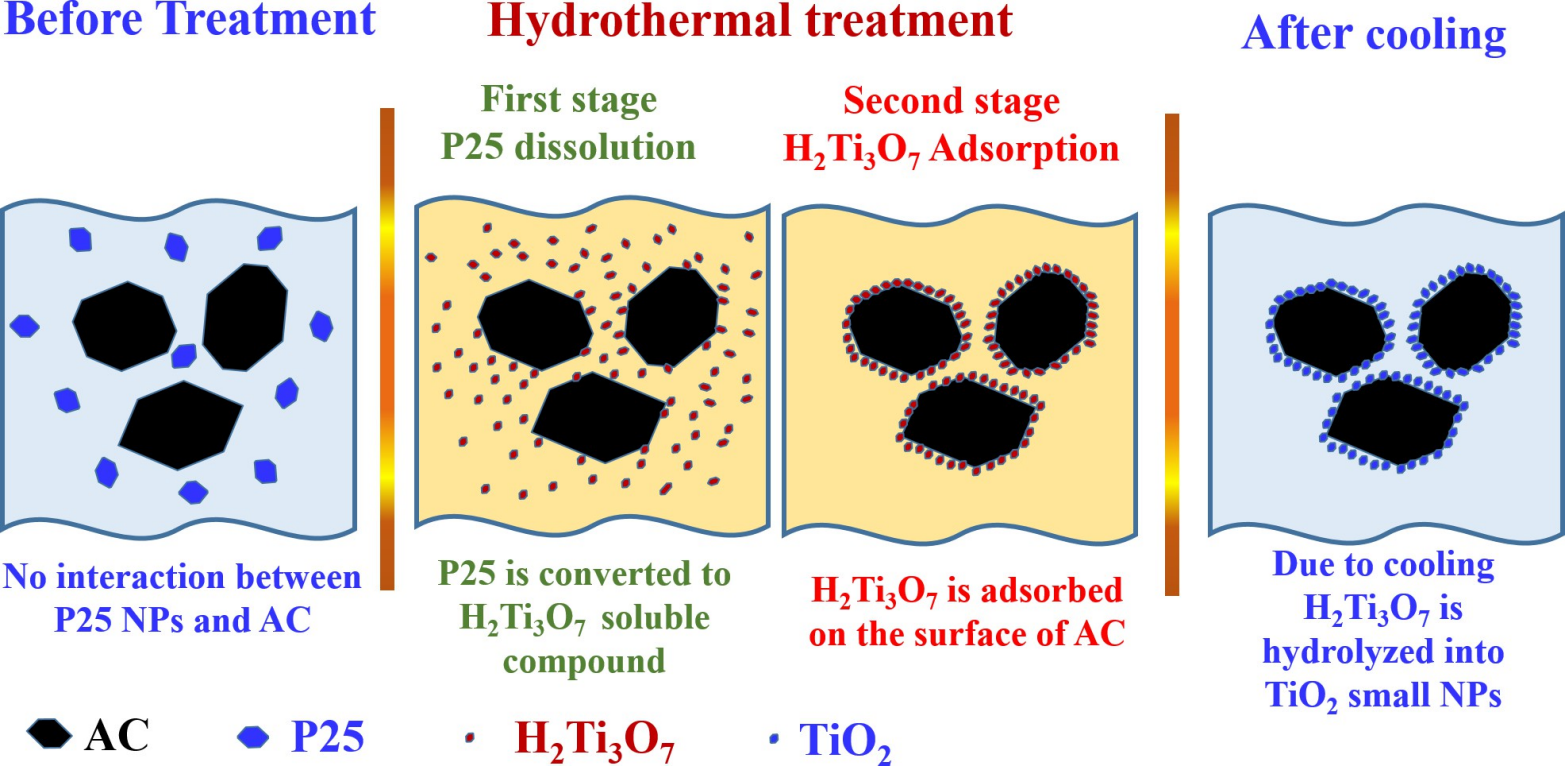
Conceptual illustration for the mechanism of TiO_2_-coated activated carbon formation using the subcritical water treatment.

According to the obtained analyses results, Fig 3 is suggested as a conceptual illustration for the formation mechanism of the prepared TiO_2_-incorporated AC. Briefly, during the hydrothermal treatment P25 are broken down to H_2_Ti_3_O_7_.*x*H_2_O nanocrystals which are adsorbed on the surface of the activated carbon. Later on, during the cooling process, H_2_Ti_3_O_7_.*x*H_2_O nanocrystals are hydrolyzed to anatase TiO_2_ thin layer covering the AC particles through a sequence of transformation series [30,34]:

$$H_{2}{\mathrm{Ti}}_{3}O_{7}\cdot xH_{2}O\to H_{2}{\mathrm{Ti}}_{3}O_{7}\cdot \to {\mathrm{TiO}}_{2}(B)\to {\mathrm{TiO}}_{2}(\mathrm{anatase})$$ (3)

Among the TiO_2_ polymorphs, the scientific research of anatase has increased due to its interesting electrochemical properties for energy application [15]. Anatase is a 700 Å thick, transparent, colorless, mesoscopic, epitaxial and low temperature stable tetragonal system. The main shape of the less orthorhombic cubic lattice is generally bipyramidal, revealing well-developed (101) faces, and this TiO_2_ phase is distinguished by the unusual capacity of combining good adsorptive and absorptive capabilities with regard to ultraviolet (UV) irradiation. Rutile type titanium dioxide, on the other hand, is a thermodynamically resistant (1.2 to 2.8 kcal.mol^-1^ more stable than anatase at temperatures between 700 and 1000°C), prismatic (unit cell is expanded beyond a cubic form), and needle-like material [25]. As a result, the manufactured TiO_2_-incorporated AC is predicted to have distinct electrochemical characteristics, similar to anatase TiO_2_.

**Fig 4:**
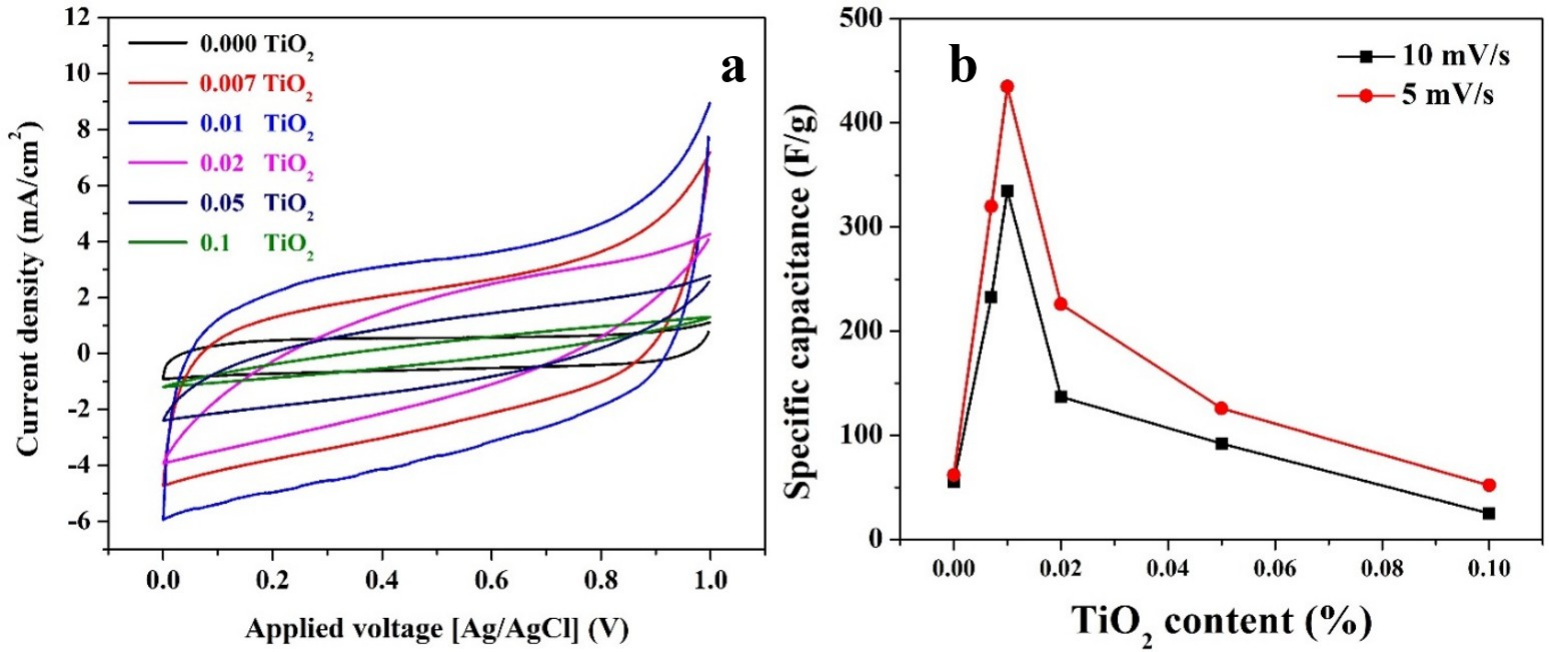
Cyclic voltammetry analyses for the prepared samples at 10 mV/s in 0.3 M NaCl solution; (a), and the influence of the TiO_2_ content on the specific capacitance; (b). The figure is in color in the online version.

Cyclic voltammetry can detect the nature of the ions electrosorption process. For instance, in the case of the pseudocapacitors, clear redox peaks appear in the cyclic voltammograms while a smooth and peaks-free cycles are obtained in the case of the physical electrosorption; EDL-based supercapacitors. Although the proposed composite contains a metal oxide, the overall behavior resembles the EDL-based supercapacitors. As shown in Fig 4A, the voltammograms for all investigated formulations do not have any redox peaks which concludes that the supercapacitance behavior for these materials can be assigned to EDLs formation; resembling the pristine AC. Fig 4B demonstrates the influence of the TiO_2_ content on the specific capacitance. As can be addressed, the highest specific capacitance could be obtained at an inorganic counterpart content of 0.01wt.% regardless the salt concentration and the applied voltage scan rate.

**Fig 5:**
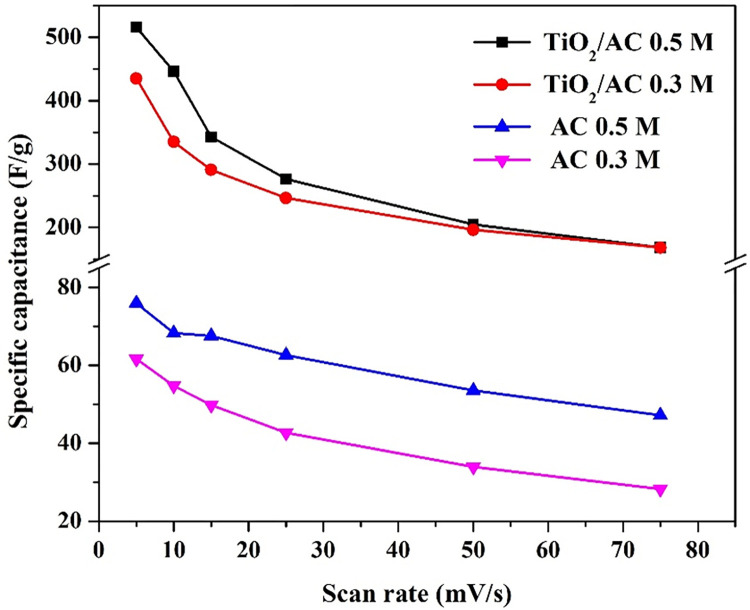
Effect of the scan rate on the specific capacitance of proposed composite (0.01 pristine TiO2) and the AC at different NaCl concentration.

Although increasing the scan rate leads to increase the generated current, the specific scan rate decreases. Slowing down the scan rate is thought to allow more electrolyte to penetrate the electrode pores and make greater contact with the internal surface of the electrode material, resulting in more charge being stored on the electrode surface and a larger measured capacitance that is closer to the intrinsic capacitance. At a greater scan rate, the electrolyte and electrode are only in contact for a short time, resulting in less charge being stored on the electrode surface and hence low capacitance [35]. Accordingly, as shown in Fig 5, increasing the scan rate led to decrease the specific capacitance. However, the most important conclusion embedded in these data is the fantastic influence of TiO_2_ incorporation on the specific capacitance. As it can be seen, at 5 mV/s, compared to the pristine AC, the specific capacitance sharply increased from 76 to 515 F/g, and from 62 to 445 F/g at 0.3 and 0.5 M NaCl, respectively due to TiO_2_ incorporation. Increasing the specific capacitance with increasing the electrolyte concentration can be attributed to mass transfer impact. In other words, high salt concentration causes to increase the mass transfer driving force (concentration difference) which is translated into increasing the number of ions reaching the electrode surface. As the specific capacitance depends mainly on the number of the adsorbed ions forming the DELs. Accordingly, it can be claimed that the maximum number of the adsorbed ions could be obtained in the case of 0.01% sample. This conclusion can be bolstered by understanding the function of each constituent in the proposed composite. AC is responsible on adsorption of the ions due to its high adsorption capacity. While, as a semiconductor, TiO_2_ enhances the capacitance characteristic. Therefore, the surface area of the uncovered AC is a critical parameter which should be in a compromise with the semiconductor content. Accordingly, it can be assumed that the surface area of the bared AC and the TiO_2_ content in the best sample represent the optimum combination. However, more increase in the metal oxide content results in decreasing the available adsorption area of the AC which negatively affects the specific capacitance of the composite. Contrarily, decreasing the amount of the TiO_2_ than the optimum value eliminates the capacitance strength of the final product.

Finally, it is believed that the proposed synthesis method in study is simple compared to the reported ones because of the following:

1. The synthesis process is based on one low temperature step. Usually, starting by solid precursors requires more than one processing step and/or high temperature treatment.
2. No additional chemicals were required.
3. Using only water as solvent.

Moreover, it is noteworthy mentioning that, the proposed procedure is novel and was not exploited before, based on our best knowledge.

Comparison with recently reported TiO_2_-based materials in term of the synthesis procedure and the obtained specific capacitance is introduced in Table 1. As shown, the prepared composite possesses very good specific capacitance. Considering the facile synthesis technique, the introduced TiO_2_-incoporated AC in this study has a privilege. Typically, the table gathers some of recently reported graphite/TiO_2_ composite prepared by different methods. According to the obtained specific capacitance, it is clear that the nano carbon matrices could distinctly improve the composite performance. For instance, TiO_2_-incoporated carbon nanofibers showed the highest specific capacitance; 691 F/g [36]. Moreover, TiO_2_/graphene oxide reveals a considerably high specific capacitance; 443 [37]. Indeed, the high surface area, the main characteristic of the nanomaterials, strongly enhances the activity due to providing numerous active sites. However, the high cost and relatively sophisticated synthesis processes are the main dilemmas facing the wide commercial application of the nanomaterials-based composites. Interestingly, although the used activated carbon in this study is widely available and cheap compared to the carbonaceous nanostructures and the proposed procedure is simple, the obtained specific capacitance (515 F/g) is close to the nano carbon-based composites.

**Table 1:** Comparison between specific capacitance values stated in the literature and the results obtained in this study.

No	Method	Name	Specific capacitance, F/g	Ref
1	Hydrothermal treatment	TiO_2_ nanorod-intercalated reduced graphene oxide (20 wt.% TiO_2_, 10 mV/s)	443	[37]
2	Electrospinning	TiO_2_/ZrO_2_ nanofibers/nitrogen co-doped activated carbon	691	[36]
3	TiO_2_ sol–gel spray	TiO_2_ sol–gel spray	104	[38]
4	Microwave-assisted ionothermal synthesis of nanostructured	Degussa-P25 TiO_2_/AC	45.9	[39]
5	Electrospinning	TiO_2_ nanofiber/activated carbon (15 wt% TiO_2_ nanofibers)	380	[40]
6	Lower temperature two-step microwave-assisted ionothermal synthesis method. (0.5 M NaCl, 10 mV/S)	Nanostructured anatase TiO_2_ /activated carbon composite	44.9	[39]
7	Microwave-assisted ionothermal synthesis method	TiO_2_ /activated carbon composite (700°C)	40.4	[41]
8	Microwave-assisted ionothermal synthesis method	TiO_2_ /activated carbon composite (800°C)	42.6	[41]
9	Microwave-assisted ionothermal synthesis method	TiO_2_ /activated carbon composite (900°C)	64.5	[41]
10	Microwave-assisted ionothermal synthesis method	AC-prisitine (700°C)	51.3	[41]
11	Microwave-assisted ionothermal synthesis method	AC-prisitine (800°C)	38	[41]
12	Microwave-assisted ionothermal synthesis method	AC-prisitine (900°C)	24.7	[41]
14	Sol–gel spray	TiO_2_ coated carbon electrode	104	[38]
15	Ultrasonication	Anatase TiO_2_-doped activated carbon fibers	182	[42]
16	Ultrasonic vibration	Energy-storage composite electrodes	155	[43]
17	Simple Hydrothermal	TiO_2_- incorporated activated carbon	515	This study

On the other hand, the table collects some AC/TiO_2_ prepared by different processes. As shown, the corresponding specific capacitances are low (mostly less than 100 F/g) compared to the introduced composite in this study. The highest values which are almost less than one third of that of the proposed combined are related to the AC/TiO_2_ composites prepared by ultrasonic treatment; 182 and 155 F/g [42,43].

## Conclusion

Titanium oxide nanoparticles-attached activated carbon can be prepared by simple hydrothermal treatment of a suspension composed of R25 titanium oxide and activated carbon at relatively high temperature. The key point is exploiting the power of the subcritical water in dissolution of TiO_2_ nanoparticles and forming a disordered colloid from hydrogen titanate nanocrystals. The later can be adsorbed on the surface of the activated carbon which results in covering the carbonaceous material by a very fine layer of anatase TiO_2_. Incorporation of TiO_2_ thin layer distinctly improves the electrosorption capacity of the final composite compared to untreated activated carbon which reflects a sharp increase in the specific capacitance. Numerically, the estimated specific capacitance is 76 to 515 F/g for the pristine and modified activated carbon, respectively at 5 mV/s in 0.5 M sodium chloride solution. Overall, this study introduces a facile strategy to synthesis valuable carbonaceous composites for several applications including supercapacitors and capacitive deionization water desalination devices. Considering utilizing solid stable precursors and only water, the introduced synthesis procedure might be recommended from the environmental point of view.

## Supporting information

S1 Graphical abstract (TIF)
