# Supplementary figures and images for: Synthesis of TiO2-incorporated activated carbon as an effective Ion electrosorption material
Source: PLoS One. 2023 Mar 23;18(3):e0282869. doi: 10.1371/journal.pone.0282869 (PMC10035829; doi:10.1371/journal.pone.0282869)

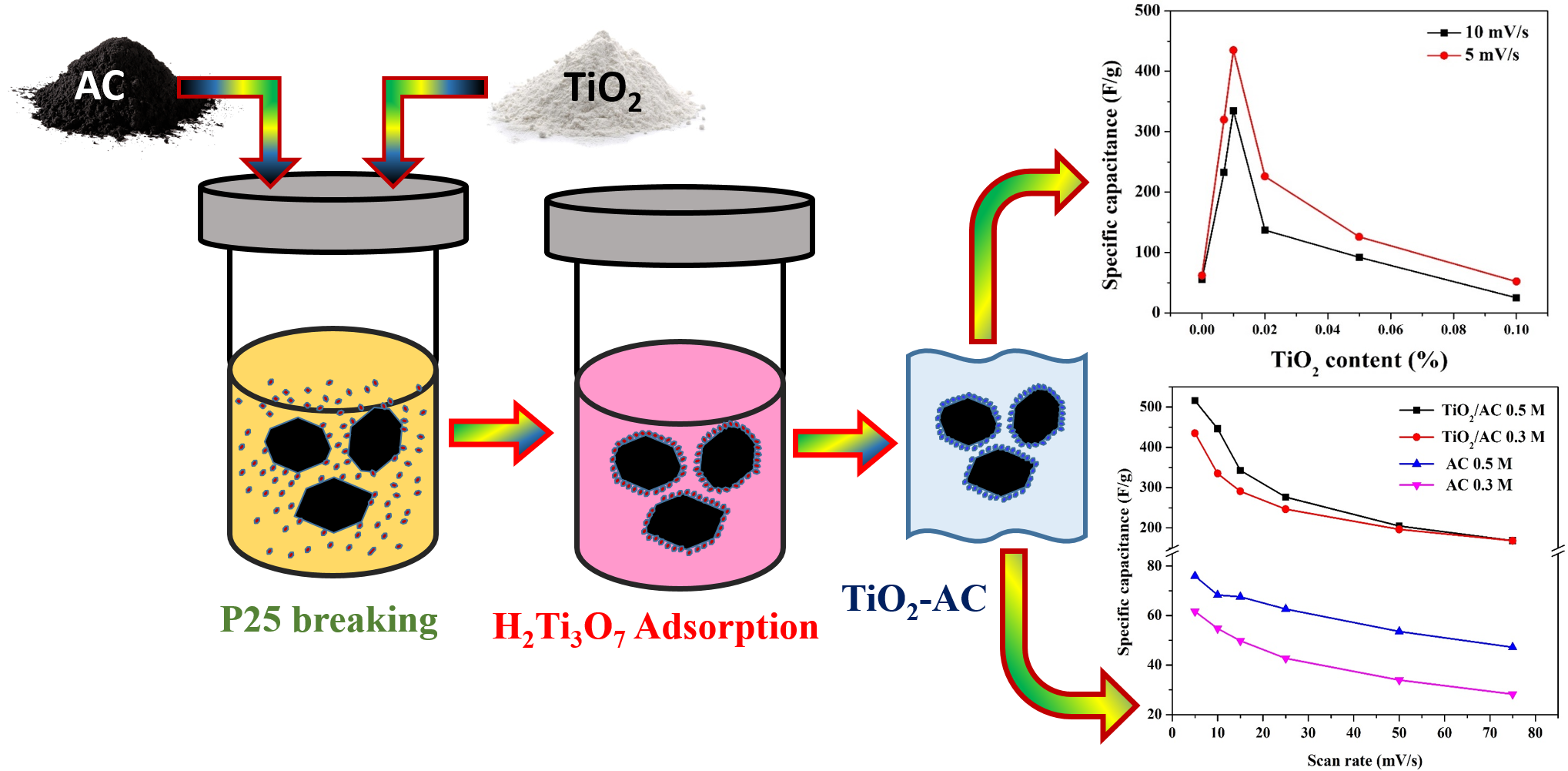

Supplement: S1 Graphical abstract — (TIF) [file pone.0282869.s001.tif]
